# The Importance of Frozen Section-Controlled Excision in Recurrent Basal Cell Carcinoma of the Eyelids

**DOI:** 10.4274/tjo.48640

**Published:** 2016-12-01

**Authors:** Berna Şahan, Ferda Çiftçi, Ferda Özkan, Vildan Öztürk

**Affiliations:** 1 Yeditepe University Faculty of Medicine, Department of Ophthalmology, İstanbul, Turkey; 2 Yeditepe University Faculty of Medicine, Department of Pathology, İstanbul, Turkey

**Keywords:** Recurrent basal cell carcinoma, frozen section, eyelid reconstruction

## Abstract

**Objectives:**

To show the importance of frozen section-controlled excision to avoid the re-recurrence of recurrent basal cell carcinoma (BCC) of the eyelids.

**Materials and Methods:**

Thirty-five cases who underwent eyelid tumor excision in different centers and were admitted to our clinic with recurrent eyelid tumors. Recurrent tumors were resected by excision 1-2 mm from the tumor’s visible margin and sent to pathology for frozen section examination. Eyelid reconstructions with flap and graft were performed after confirming that the surgical margins were negative.

**Results:**

Twenty-one (60%) of our patients were male and 14 (40%) were female. Median age of our group was 63.4±14.2 years. Excision and sending the excised material for frozen section control was performed once for 11 patients, twice for 12 patients, 3 times for 8 patients and 4 times for 4 patients to confirm that the surgical margins were clean. All pathology samples were reported as BCC. All patients had eyelid reconstruction with flap and graft. Recurrence was detected in 2 patients (5.7%) during 1 to 8 years (mean 4.3 years) of follow-up and those patients were reoperated; no recurrence was detected in the remaining 33 patients (94.3%).

**Conclusion:**

Frozen section control can provide low re-recurrence rate in patients with recurrent BCC of the eyelids.

## INTRODUCTION

Basal cell carcinoma (BCC) comprises approximately 90% of malignant tumors on and around the eyelid.^1^ In Turkey this rate has been reported as 70-95.5%.^2,3,4,5,6^ Prolonged sun exposure, light skin complexion, advanced age, and diseases like Xeroderma pigmentosum and Gorlin syndrome are among the known risk factors for BCC.^7^

The most common histopathologic subtype of BCC is the nodular type.^8^ Rodent ulcers, which form as a result of a nodule with central elevation and overlying ulceration, are seen in this type. The morpheaform type of BCC is a more aggressive tumor and may simulate chronic blepharitis clinically.^9^

In the periocular region, BCC occurs most often in the lower eyelid, followed by the inner canthus, upper eyelid and outer canthus.^10^ BCC generally progresses slowly and very rarely metastasizes.^11^ Local spread to surrounding tissues is clinically significant. Tissues which may be affected include the conjunctiva, cornea, orbit, paranasal sinuses, nasal cavity and central nervous system.^12^

Frozen section is a technique which ensures clean surgical margins during excision. In this procedure, after excising the mass, its anatomic position is mapped on paper and the mass is sent to pathology for frozen section examination. If carcinoma cells are found at the surgical margins, the excision area is enlarged and frozen section control is repeated. This process is repeated until the surgical margins are clean.^13^

Surgery excision is considered the gold standard in BCC therapy.^14^ Surgical techniques like Mohs micrographic surgery and frozen section can be used to minimize postoperative recurrence. Postoperative recurrence of primary BCC has been reported at rates of 1.7% in the frozen section group and 1.6% in a Mohs micrographic surgery group.^13,15^ Although both of these techniques result in similar recurrence rates, Mohs micrographic surgery is more difficult and costly to perform.16 The aim of the present study was to report the surgical outcomes of patients who presented to our clinic with recurrent periocular BCC after primary excision and underwent frozen section controlled excision to prevent further recurrence.

## MATERIALS AND METHODS

The records of all patients who had previously undergone a primary surgery for periocular BCC and who later underwent frozen section-controlled excision in our clinic due to recurrence between 2007 and 2015 were analyzed retrospectively. Preoperatively, all patients’ initial histologic diagnosis was reported as BCC. The records of 37 patients met these criteria; 2 patients were excluded from the study due to inadequate follow-up time. Thirty-five eyes of 35 patients followed regularly for at least 1 year were included in the study.

Patients were evaluated in terms of age, gender, location of the mass, how many rounds of intraoperative frozen section were performed, surgery duration, mass histopathology (noduloulcerative type or morpheaform type), spread to surrounding tissues, reconstructive procedures used, presence of new recurrence, time and location of new recurrence, and follow-up time.

**Figure 1:**
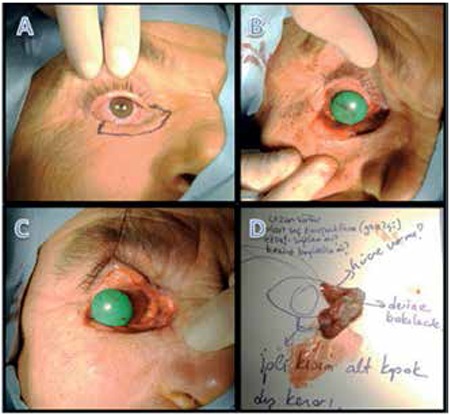
A 54-year-old male patient with mass in the left lower eyelid and medial canthal area: preoperative marking of mass margins (A); appearance after intraoperative frozen section-controlled excision (B,C); appearance of mass while sending for frozen section examination (D)

All operations were performed by the same surgeon (F.Ç.). After marking the margins of the BCC with a sterile pen, local anesthesia was injected (2% lidocaine with 1/10.000 adrenaline). The area of excision extended 1-2 mm beyond the apparent mass margin; the mass was mapped on paper, then sent to pathology for frozen section examination (Figure 1). The excision area was enlarged and frozen section was repeated until the surgical margins were clean on examination.

Specimens for frozen section were frozen to -22 °C within 10 minutes in the Shandon Cryotome SME Cryostat (Thermo Fisher Scientific, Inc., Waltham, MA, USA) and 5-micron-thick sections of the surgical margin were stained for 3 minutes with hematoxylin-eosin (H&E) stain. All surgical margins were evaluated by pathologist and reports were issued. The total examination time, including all procedures, varied between 15 and 20 minutes for each sample.

Eyelid reconstruction procedures were performed after the results of pathologic examination confirmed the surgical margins were clean. Reconstructive procedures were chosen based on the size, location and shape of the defect and the anatomic structures involved.

During reconstruction for partial lower eyelid defects, the posterior lamella was created from an ipsilateral upper eyelid tarsoconjunctival flap (modified Hughes method), while contralateral upper eyelid tarsoconjunctival grafts were used for larger defects. The reconstruction procedure was completed by creating the anterior lamella using a cheek advancement or rotation flap. For partial upper eyelid defects, reconstruction was done using an ipsilateral tarsoconjunctival transposition flap (tarsal rotation flap) to create the posterior lamella and an ipsilateral upper eyelid transposition flap or contralateral upper eyelid free graft for the anterior lamella. For larger upper eyelid defects, reconstruction was done using a lower eyelid tarsoconjunctival flap and free muscle-skin graft for the anterior lamella or by one-step reconstruction (contralateral upper eyelid tarsoconjunctival graft and local muscle-skin flap for the anterior lamella). Patients with upper eyelid defects and excision of the medial canthal area underwent reconstruction by tarsal rotational flap and glabellar rotation flap recruited from the forehead.

No complications occurred in any of the patients postoperatively. Patients were followed at 6-month intervals.

## RESULTS

Mean age of the 35 patients who were diagnosed with recurrent BCC and underwent frozen section controlled excision was 63.4±14.2 years (range, 35-83 years). Twenty-one (60%) of the patients were female, 14 (40%) were male. BCC was located on the lower eyelid in 26 patients (74.3%), upper eyelid in 4 (11.4%) and upper eyelid/medial canthal region in 5 patients (14.3%).

Frozen section control was performed once in 11 patients, twice in 12 patients, 3 times in 8 patients and 4 times in 4 patients in order to achieve clean surgical margins. Time required for the frozen section procedure ranged from 15-20 minutes for all samples. Definite pathologic examination results were reported as morpheaform BCC in 2 cases (5.7%) and as noduloulcerative BCC in the remaining 33 cases (94.3%) (Table 1). Lacrimal system involvement was noted in one patient whose mass was in the upper eyelid/medial canthal region; the lacrimal system and canaliculi were included in the excision area (Figure 1).

**Figure 2:**
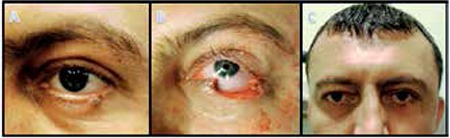
A 35-year-old male patient with mass of the lower eyelid: preoperative appearance (A); appearance after 3 rounds of intraoperative frozen section controlled excision (B); postoperative 1 year appearance after reconstruction by ipsilateral tarsoconjunctival flap and cheek muscle-skin advancement (C)

**Figure 3:**
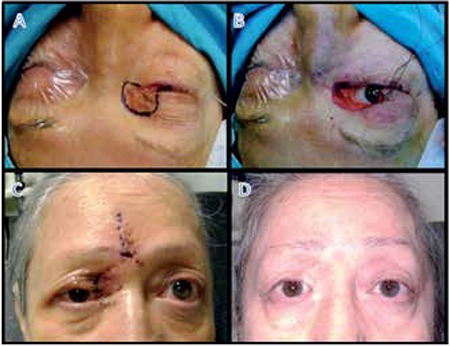
A 65-year-old female patient: preoperative marking showing planned excision area (A); appearance of the excision area related to the lacrimal system after 3 rounds of frozen section controlled excision (B); appearance at 1 week after reconstruction using tarsal rotational flap and glabellar skin flap (C); appearance at postoperative 3 years (D)

In all patients, primary repair was inadequate to reconstruct the eyelid defects resulting from surgical excision. Therefore, graft and flap reconstruction was done in all patients (Figures 2, 3).

Modified Hughes procedure and cheek muscle-skin advancement flap was performed in 14 patients in whom more than 50% of the lower eyelid was excised and primary closure could not be performed (Figure 2). For the 5 patients with full lower eyelid defect, the posterior lamella was formed by a tarsoconjunctival graft taken from the contralateral upper eyelid, while the anterior lamella was formed using a cheek transposition or rotation flap.

For the 9 patients whose upper eyelid defects could not be repaired by primary closure or had 50-75% of the upper eyelid excised, reconstruction was done using an ipsilateral tarsal rotational flap to create the posterior lamella and an ipsilateral upper eyelid advancement or contralateral upper eyelid free graft for the anterior lamella. Three patients with defects greater than 75% of the upper eyelid after excision underwent reconstruction using either lower eyelid tarsoconjunctival flap and free muscle-skin graft or one-step reconstruction.

Of the 4 patients whose mass was located in the upper eyelid/medial canthal region, lacrimal system involvement was discovered intraoperatively in 1 patient and the excision area was expanded to include the lacrimal sac and canaliculi. During reconstruction for these 4 patients, the posterior lamella was formed using ipsilateral tarsal rotational flap and the anterior lamella of the upper eyelid and canthal region was created with a glabellar rotation flap.

A cosmetically acceptable outcome was achieved in all cases. Patients were followed at 6-month intervals. Recurrence occured in 2 patients (5.7%) during the postoperative follow-up period of 1-8 years (mean: 4.3±2.1 years), at postoperative 1 year in a patient with total lower eyelid involvement and at postoperative 7 months in a patient with medial canthal region and lacrimal system involvement. Definitive pathology was reported as morpheaform BCC for both of the patients with recurrence.

## DISCUSSION

BCC is the most common malign neoplasm of the periocular region. About 95% of patients with BCC are between 40 and 79 years of age. Its slow progression and spread to surrounding tissues conjunctiva, cornea, orbit, paranasal sinuses, nasal cavity and central nervous system) are clinically significant.^11,12^ Spread of the tumor into surrounding tissues makes complete excision and reconstruction a challenge.

Risk factors for recurrence in BCC include previous recurrence of the tumor, location in the medial canthal region,^17^ morpheaform type^18^ and large tumor size. Mohs^19^ reported a cure rate of 80% in patients with tumors larger than 3 cm, whereas the cure rate for smaller tumors was 99.4%.

Nonsurgical treatment options for BCC include cryotherapy, radiotherapy, photodynamic therapy, curettage and electrodissection, and topical immunomodulators such as topical 5-fluorouracil and imiquimod. However, surgical excision is accepted as the definitive treatment for BCC.^14^ Recurrence rates after BCC excision and primary repair without performing Mohs micrographic surgery or frozen section controlled surgery were reported as 64% by Downes et al.,^20^ 50% by Older et al.,^21^ 26% by Doxanas et al.^22^ and in Turkey, 8% by Günalp and Akbaş8 and 16.7% by Yalçın Tök et al.^23^ Variations in amount of tissue excised and follow-up times contribute to the differences in these reported rates.

Recurrence rates after frozen section controlled excision were reported as 1.7% by Gayre et al.,^13^ 4% by Nemet et al.,^10^ 0.7% by Wong et al.,^24^ 0.26% by Ho et al.^25^ and 1.3% by Gill et al.,^26^ while no recurrence was observed by Conway et al.^27^ after 5 years, by Taherian et al.^28^ after 38 months or by Akbaş Kocaoğlu et al.^29^ in Turkey after 18.7 months of follow-up.

Among patients with recurrent BCC, new recurrence occurred after frozen section-controlled excision in 4.4% of 21 patients studied by Older et al.,^21^ 3.8% of 26 patients for Ho et al.^25^ and 4.8% of 21 patients in a study by Giordano Resti et al.^30^

This demonstrates that the recurrence rate is higher in recurrent BCC than in primary BCC. Consistent with these other studies, recurrence occurred in 2 patients (5.7%) in the present study during the follow-up period. Evaluation of recurrent BCC cases in the literature reveals that tumors of the morpheaform subtype and those located in the medial canthus are particularly prone to recurrence.^25,30^ In our series, both recurrent tumors were of the morpheaform type; one was located in the medial canthus area, while the other showed total lower eyelid involvement.

The Mohs micrographic surgery is currently considered the most reliable intraoperative method for minimizing the recurrence rate of BCC.^31^ In the procedure, tissue blocks which are 5-10 mm2 and 2-4 mm thick are excised in a lamellar fashion until the surgical margins are proven to be clear. The recurrence rate after Mohs micrographic surgery has been reported as 2% over a 5-year follow-up period, with this rate increasing to 3-20% in patients with previous recurrence.^15,32,33,34^ However, the procedure cannot be performed in many clinics in Turkey and abroad due to the cost and need for an experienced pathologist.^16^ In recurrent BCC cases, frozen section controlled excision and Mohs micrographic surgery have comparable postoperative recurrence rates.

All patients in the present study presented to our clinic with recurrent BCC, and treatment with frozen section controlled excision was chosen in order to reduce the risk of possible re-recurrence. The first excision was done 1-2 mm beyond the visible tumor margin. The number of rounds of frozen section control required for the pathologist to intraoperatively confirm clean surgical margins was 1 in 11 patients, 2 in 12 patients, 3 in 8 patients and 4 in 4 patients. These excisions resulted in an excision area that was several times larger than the apparent size of the tumor preoperatively. Graft and flap eyelid reconstruction was performed in all cases. It is well known that BCC can extensively invade surrounding tissues and that excising an area much larger than the clinically visible tumor may be necessary, especially in cases of recurrence. In the present study, frozen section controlled excision both ensured that enough tissue was removed to achieve clean surgical margins and allowed the labor intensive reconstruction procedures to be conducted with confidence knowing that the surgical margins were clean.

During the follow-up period of mean 4.3 years, 2 patients (5.7%) experienced recurrence, 1 with total lower eyelid involvement and 1 with a tumor in the upper eyelid/medial canthal region with lacrimal gland involvement, and were reoperated; recurrence was not detected in the other 33 patients (94.3%). All patients in our series presented with recurrent BCC; therefore, frozen section controlled excision was chosen in order to minimize the risk of new recurrence postoperatively. Excision was initially performed 1-2 mm beyond the visible tumor margins and the excision area was enlarged until clean surgical margins were confirmed. Traditionally, in BCC surgery the excision area includes 3-4 mm of healthy tissue.^25^ Furthermore, it is known that inadequate excision increases recurrence. However, excessive tissue removal makes reconstructive procedures more challenging and may be an obstacle to achieving a cosmetically acceptable outcome. It is therefore considered adequate to begin excising 1-2 mm beyond the visible lesion in surgeries performed with intraoperative frozen section control. Using the frozen section control procedure in our patients, we ensured clean surgical margins while minimizing tissue excision and achieved cosmetically acceptable results after reconstruction (Figures 2, 3).

Intraoperative frozen section control extends surgery times, increases costs and requires an experience pathologist to be present at the medical center where the surgery is performed. In recurrent BCC, which has a higher recurrence rate than primary BCC, excision with frozen section control may lower the incidence of recurrence for these patients.

### Ethics

Ethics Committee Approval: A retrospective study, Informed Consent: It was taken.

Peer-review: Externally peer-reviewed.

## Figures and Tables

**Table 1:**
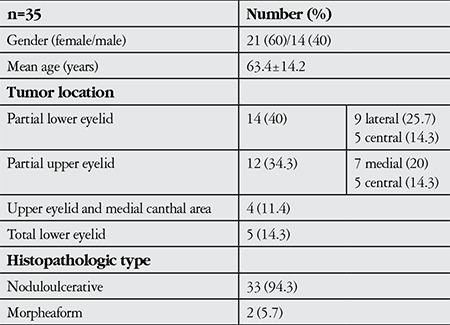
Patients’ demographic data and tumoral anatomic location and histologic type

## References

[1] Donaldson MJ, Sullivan TJ, Whitehead KJ, Williamson RM (2002). Squamous cell carcinoma of the eyelids. Br J Ophthalmol..

[2] Demir CY, Köhle Ü (2003). Periorbital bölge malign cilt tümörleri: retrospektif çalışma. Fırat Tıp Dergisi..

[3] Özkılıç E, Peksayar G (2003). Epidemiologic investigation of eyelid tumors. Turk J Ophthalmol..

[4] Soysal H, Albayrak A (2001). Primary malignant tumors of the eyelids. Turk J Ophthalmol..

[5] Taşkıran Çömez A, Akçay L, Özgür Ö, Karadağ O, Doğan ÖK (2007). Histopathological and epidemiological evaluation of eyelid masses. Turk J Ophthalmol..

[6] Gundogan FC, Yolcu U, Tas A, Sahin OF, Uzun S, Cermik H, Ozaydin S, Ilhan A, Altun S, Ozturk M, Sahin F, Erdem U (2015). Eyelid tumors: clinical data from an eye center in Ankara, Turkey. Asian Pac J Cancer Prev..

[7] Dean-Ferrer A, Arroyo Rodriguez S, Calderon-Polanco J, Alamillos Granados FJ, Poblet E (2009). Basal cell nevus syndrome: clinical and genetic diagnosis. Oral Maxillofac Surg..

[8] Günalp İ, Akbaş F (1996). Göz kapağının bazal hücreli karsinomu: 1100 olguda klinik bulgular ve tedavi yaklaşımları. MN Oftalmoloji..

[9] Loeffler M, Hornblass A (1990). Characteristics and behavior of eyelid carcinoma (basal cell, squamous cell sebaceous gland, and malignant melanoma). Ophthalmic Surg..

[10] Nemet AY, Deckel Y, Martin PA, Kourt G, Chilov M, Sharma V, Benger R (2006). Management of periocular basal and squamous cell carcinoma: a series of 485 cases. Am J Ophthalmol..

[11] Prabhakaran VC, Gupta A, Huilgol SC, Selva D (2007). Basal cell carcinoma of the eyelids. Compr Ophthalmol Update..

[12] Weber RS, Lippman SM, McNeese MD (1990). Advanced basal and squamous cell carcinomas of the skin of the head and neck. Cancer Treat Res..

[13] Gayre GS, Hybarger CP, Mannor G, Meecham W, Delfanti JB, Mizono GS, Guerry TL, Chien JS, Sooy CD, Anooshian R, Simonds R, Pietila KA, Smith DW, Dayhoff DA, Engman E, Lacy J (2009). Outcomes of excision of 1750 eyelid and periocular skin basal cell and squamous cell carcinomas by modified en face frozen section margin-controlled technique. Int Ophthalmol Clin..

[14] Cook BE, Bartley GB (2001). Treatment options and future prospects for the management of eyelid malignancies: an evidence-based update. Ophthalmology..

[15] Litwin AS, Rytina E, Ha T, Rene C, Woodruff SA (2013). Management of periocular basal cell carcinoma by Mohs micrographic surgery. J Dermatolog Treat..

[16] Hamada S, Kersey T, Thaller VT (2005). Eyelid basal cell carcinoma: non-Mohs excision, repair, and outcome. Br J Ophthalmol..

[17] Abe M, Ohnishi Y, Hara Y, Shinoda Y, Jingu K (1983). Malignant tumor of the eyelid--clinical survey during 22-year period. Jpn J Ophthalmol..

[18] Wolf DJ, Zitelli JA (1987). Surgical margins for basal cell carcinoma. Arch Dermatol..

[19] Mohs FE (1986). Micrographic surgery for the microscopically controlled excision of eyelid cancers. Arch Ophthalmol..

[20] Downes RN, Walker NP, Collin JR (1990). Micrographic (MOHS’) surgery in the management of periocular basal cell epitheliomas. Eye (Lond)..

[21] Older JJ, Quickert MH, Beard C (1975). Surgical removal of basal cell carcinoma of the eyelids utilizing frozen section control. Trans Sect Ophthalmol Am Acad Ophthalmol Otolaryngol..

[22] Doxanas MT, Green WR, Iliff CE (1981). Factors in the successful surgical management of basal cell carcinoma of the eyelids. Am J Ophthalmol..

[23] Yalçın Tök Ö, Akbaş Kocaoğlu F, Örnek F (2010). Surgery of primary basal cell carcinoma with frozen section controlled excision. Turk J Ophthalmol..

[24] Wong VA, Marshall JA, Whitehead KJ, Williamson RM, Sullivan TJ (2002). Management of periocular basal cell carcinoma with modified en face frozen section controlled excision. Ophthal Plast Reconstr Surg..

[25] Ho SF, Brown L, Bamford M, Sampath R, Burns J (2013). 5 years review of periocular basal cell carcinoma and proposed follow-up protocol. Eye (Lond)..

[26] Gill HS, Moscato EE, Seiff SR (2014). Eyelid margin basal cell carcinoma managed with full-thickness en-face frozen section histopathology. Ophthal Plast Reconstr Surg..

[27] Conway RM, Themel S, Holbach LM (2004). Surgery for primary basal cell carcinoma including the eyelid margins with intraoperative frozen section control: comparative interventional study with a minimum clinical follow up of 5 years. Br J Ophthalmol..

[28] Taherian K, Shekarchian M, Atkinson PL (2007). Surgical excision of periocular basal cell carcinomas. Indian J Ophthalmol..

[29] Akbaş Kocaoğlu F, Yalçın Tök Ö, Burcu A, Örnek F (2010). Kapak malign tümörlerinde dondurulmuş kesit denetimli eksizyon ve kapak rekonstrüksiyonu. MN Oftalmoloji.

[30] Giordano Resti A, Sacconi R, Baccelli N, Bandello F (2014). Outcome of 110 basal cell carcinomas of the eyelid treated with frozen section-controlled excision: mean follow-up over 5 years. Eur J Ophthalmol..

[31] Malhotra R, Huilgol SC, Huynh NT, Selva D (2004). The Australian Mohs database, part II: periocular basal cell carcinoma outcome at 5-year follow-up. Ophthalmology..

[32] Mohs FE (1986). Micrographic surgery for the microscopically controlled excision of eyelid cancer: history and development. Adv Ophthalmic Plast Reconstr Surg..

[33] Robins P, Rodriguez-Sains R, Rabinovitz H, Rigel D (1985). Mohs surgery for periocular basal cell carcinomas. J Dermatol Surg Oncol..

[34] Lang PG (1989). Mohs micrographic surgery. Fresh-tissue technique. Dermatol Clin..

